# *SmuMYB113* is the determinant of fruit color in pepino (*Solanum muricatum*)

**DOI:** 10.3389/fpls.2024.1408202

**Published:** 2024-06-20

**Authors:** Marcela Martinez-Sanchez, Donald A. Hunter, Ali Saei, Christelle M. Andre, Erika Varkonyi-Gasic, Glen Clark, Emma Barry, Andrew C. Allan

**Affiliations:** ^1^ The New Zealand Institute for Plant and Food Research Limited (Plant & Food Research) Mt Albert, Auckland, New Zealand; ^2^ School of Biological Sciences, University of Auckland, Auckland, New Zealand; ^3^ The New Zealand Institute for Plant and Food Research Limited (Plant & Food Research), Palmerston North, New Zealand; ^4^ Grasslands Research Centre, AgResearch Limited, Palmerston North, New Zealand

**Keywords:** plant model, *Solanum muricatum*, anthocyanins, MYB, transcriptomics

## Abstract

Pepino (*Solanum muricatum*) is an herbaceous crop phylogenetically related to tomato and potato. Pepino fruit vary in color, size and shape, and are eaten fresh. In this study, we use pepino as a fruit model to understand the transcriptional regulatory mechanisms controlling fruit quality. To identify the key genes involved in anthocyanin biosynthesis in pepino, two genotypes were studied that contrasted in foliar and fruit pigmentation. Anthocyanin profiles were analyzed, as well as the expression of genes that encode enzymes for anthocyanin biosynthesis and transcriptional regulators using both RNA-seq and quantitative PCR. The differential expression of the transcription factor genes R2R3 MYB *SmuMYB113* and R3MYB *SmuATV* suggested their association with purple skin and foliage phenotype. Functional analysis of these genes in both tobacco and pepino showed that SmuMYB113 activates anthocyanins, while SmuATV suppresses anthocyanin accumulation. However, despite elevated expression in all tissues, *SmuMYB113* does not significantly elevate flesh pigmentation, suggesting a strong repressive background in fruit flesh tissue. These results will aid understanding of the differential regulation controlling fruit quality aspects between skin and flesh in other fruiting species.

## Introduction

Pepino (*Solanum muricatum* Aiton, 2n=24), is an herbaceous crop native to the High Andes region of South America (Anderson et al., 1996). This crop is in the same genus as tomato (*S. lycopersicum* L.) and potato (*S. tuberosum* L.) and is phylogenetically closely related to other major solanaceous fruit crops such as pepper (*Capsicum annuum* L.) and eggplant (*Solanum melongena* L.) (Spooner et al., 1993; Rodríguez-Burruezo et al., 2011; Sarkinen et al., 2013). However, it has a number of advantageous characteristics, including propagation and growing practices. Pepino plants are easy to grow and propagate, have a relatively short life cycle, simple genetics (diploid), and a recently sequenced genome (Song et al., 2022). Pepino has a tendency to produce parthenocarpic fruit (Prohens et al., 2005), and the fruit need over 90 days to reach rich full ripeness. Pepino plants are sensitive to high temperatures during pollination, which can significantly reduce fruit set (Burge, 1989) and affect ripening, fruit quality and taste (Rodríguez-Burruezo et al., 2011).

Pepino fruit can be round, ellipsoid or elongated (Herraiz et al., 2015), and have attractive characteristics for consumers, such as brightly colored skin with stripes in some genotypes, intense aroma and a yellow juicy flesh with a mild sweet taste (Rodríguez-Burruezo et al., 2011; Herraiz et al., 2016). Fruit are generally consumed when fully ripe in the same way as melon, as a dessert fruit, although they are notably less sweet (Prohens et al., 2005). Another use for the un-ripe fruit is to cut and use in a similar way to cucumber. The pepino fruit is recognized for its beneficial attributes for human health (Hsu et al., 2011; (Shathish and Guruvayoorappan, 2014). The relatively large variation in appearance, anthocyanin concentration, soluble solids content, acidity, and vitamin C content (Rodríguez-Burruezo et al., 2011 (Prohens et al., 2003), Herraiz et al., 2016), suggests that improvement can be made via selection and breeding (Rodríguez-Burruezo et al., 2011), with breeding goals such as enhanced yield, reduced ripening time, improved heat tolerance and increased fruit quality.

Anthocyanins are flavonoids synthesized by plants and along with other pigments such as betalains and carotenoids, are responsible for the red to blue color in leaves, flowers and fruits (Holton and Cornish, 1995; Grotewold, 2006; Petroni and Tonelli, 2011). They help attract pollinators and seed distributors (Davies et al., 2012) and also play a critical role in protecting the plant against abiotic stresses including drought (Castellarin et al., 2007; André et al., 2009), UV radiation and cold temperatures (Christie et al., 1994; Sarma and Sharma, 1999). In addition, anthocyanins have been identified as powerful antioxidants and anti-inflammatory agents in the human body (Kähkönen and Heinonen, 2003; Miguel, 2011).

Anthocyanin biosynthesis and regulatory pathways have been extensively characterized in many plant species (Holton and Cornish, 1995; Petroni and Tonelli, 2011). The co-ordinated expression of what has been termed ‘early’ biosynthetic genes: chalcone synthase (CHS), chalcone isomerase (CHI) and flavone3-hydroxylase (F3H); and what has been termed ‘late’ biosynthetic genes encoding flavonoid 3'5'-hydroxylase ((F3'5'H), dihydroflavonol 4-reductase (DFR), anthocyanidin synthase (ANS), flavonoid 3-*o*-glucosyltransferase (3GT), anthocyanin rhamnosyltransferase (RT), anthocyanin acyltransferase (AAC), flavonoid 5-O-glucosyltransferasese (5GT), and glutathione *S*-transferase (GST) generate anthocyanins. This transcriptional regulation is by DNA-binding R2R3 MYB transcription factors (TFs), MYC-like basic helix-loop-helix (bHLH) TFs, and WD40-repeat proteins in the MYB-bHLH-WD40 (MBW) complex (Baudry et al., 2004; Allan et al., 2008; Wang et al., 2020).

Expression of the genes encoding the MBW complex is activated or repressed by environmental and developmental factors which then drive expression of downstream targets (Allan and Espley, 2018). It is often a MYB transcription factor (TF) that is the limiting factor in this response. The R2R3 MYBs participate in regulating both early and late genes of the anthocyanin biosynthetic pathway (Schwinn et al., 2006; Dubos et al., 2010). These activators (often MYB TFs sub-group 6) have been characterized in many species including *Arabidopsis* (Borevitz et al., 2000; Gonzalez et al., 2008), and many others including maize (Paz-Ares et al., 1987), potato (Jung et al., 2005, 2009; Zhang et al., 2009), tomato (Mathews et al., 2003), pepper (Borovsky et al., 2004), sweet potato (Chu et al., 2013), petunia (Quattrocchio et al., 1999), grapevine (Kobayashi et al., 2005; Walker et al., 2007; Deluc et al., 2008), Bayberry (Liu et al., 2013), and apple (Takos et al., 2006; Ban et al., 2007; Espley et al., 2007). However, there is apparently strong repression or restrictions on these activators, as certain tissues accumulate anthocyanins while other tissues do not.

In contrast, R2R3 MYB TFs of subgroup 4 have been identified as negative regulators of the anthocyanin biosynthetic pathway (LaFountain and Yuan, 2021) in several species, including strawberry (Aharoni et al., 2001), snapdragon (Tamagnone et al., 1998), apple (Lin-Wang et al., 2011), grapevine (Cavallini et al., 2015), Arabidopsis (Jin et al., 2000), petunia (Albert et al., 2011; Albert et al., 2014a) and ginkgo (Xu et al., 2014). Subgroup 4 repressors actively repress transcription of their target genes, mediated by repressive motifs (e.g. ERF Amphiphilic Repression – EAR, and TLLLFR) within their C-termini (Tamagnone et al., 1998; Aharoni et al., 2001; Matsui et al., 2008). These R2R3 MYB repressors are co-repressors acting within the MBW complex, binding to the bHLH proteins, and can convert an MBW complex into one that inhibits transcription (Matsui et al., 2008; Albert et al., 2014a, 2014b).

R3 MYBs contain a single imperfect repeat and are therefore classed within the 1R-MYB-like subclass. However, these MYBs are unable to bind DNA directly, as they lack a transcriptional activation domain present in R2R3 MYB TFs, and therefore these R3 MYBs affect transcription of their target genes with other partners such as bHLHs (Du et al., 2015). The R3 repeat allows the R3 MYB to interact with bHLH partners, and several have been shown to act as inhibitors of transcriptional regulation, by competing with R2R3 MYBs for the MBW complex (Matsui et al., 2008; Xu et al., 2015). In Arabidopsis, AtCPC competes with the activator MYB, AtPAP1, for the formation of the MBW complex (Zhu et al., 2009). In tomato, the R3 MYB SlATV inhibits the expression of the structural genes responsible for anthocyanin biosynthesis, leading to a repression of anthocyanin production (Cao et al., 2017).

Anthocyanin biosynthesis and regulation has been widely characterized in solanaceous species (Albert et al., 2011; Albert et al., 2014a; Kiferle et al., 2015; Montefiori et al., 2015; Liu et al., 2016, 2018; Colanero et al., 2020; Sun et al., 2020; Yan et al., 2020; Zhou et al., 2022). However, less is known about anthocyanin production in pepino, although key genes have been reported in the recently published genome (Song et al., 2022). The profile of phenolic acids, flavonoids, and anthocyanins in aqueous extracts of pepino fruit was reported (Hsu et al., 2011) with (Wu et al., 2013) identifying eight phenolic compounds and one flavonoid in the fruit. The diversity of plant color within pepino fruit, including green, yellow and purple-skinned varieties, as well as its close phylogenetic relationship to potato and tomato, makes pepino an ideal candidate for further investigation and modeling of anthocyanin biosynthesis in the Solanaceae, as well as fruiting plant crops outside the Solanaceae family. In pepino, the variation in phenolic and polyphenolic compounds (including flavonoids and total anthocyanins) has been studied (Anderson et al., 1987; Hsu et al., 2011; Herraiz et al., 2016; Hsu et al., 2018). Also, by comparing wild pepino and a cultivated pepino, Song et al. (2022) used RNAseq aligned to their constructed genome, to study the anthocyanin pathway and its regulation. Here, we studied pepino lines with different fruit and foliar pigmentation, identified the anthocyanin profile present in pepino skin, and used transcriptomics to determine the controlling transcription factors for anthocyanin biosynthesis. Transient and stable transformation showed that pepino R2R3 MYB113 (AN1-like) is a key transcriptional activator for pepino anthocyanin accumulation. However, a strong repressive background is suggested in the fruit flesh, which prevents anthocyanin accumulation in this tissue.

## Materials and methods

### Plant material

A purple-skinned pepino (*S. muricatum)* genotype, termed Purple Selection (PS, purple skin and yellow flesh) and a yellow-skinned pepino selection, Yellow Selection (YS, yellow skin and yellow flesh) were propagated by cuttings in a greenhouse at Plant & Food Research, Auckland, New Zealand. After 56 days, rooted cuttings were transferred into 30-L pots and grown in a greenhouse at Plant & Food Research, Pukekohe, New Zealand, at ambient conditions. Ten to thirteen pepino fruit from separate plants were collected at 20, 34, 48 and 73 days after flowering (DAF), and pooled from at least three plants (each stage of fruit development had three biological replicates). Skin tissue was carefully separated from flesh tissue, with any residual flesh tissue removed (pepino has a very strong skin layer) and frozen separately in liquid nitrogen and stored at −80°C.

For functional gene testing, *Nicotiana tabacum* (tobacco) and pepino plants were grown under glasshouse conditions between 20 to 24°C using natural daylight with extension to 16 h at Plant & Food Research, Auckland, New Zealand.

### Anthocyanin identification and quantification

Pepino extracts were analyzed with a Waters Acquity® Ultra Performance Liquid Chromatography (UPLC) system (Milford, MA, USA) equipped with a photodiode array detector (PDA) and a single quadrupole mass spectrometer (QDa, Waters, MA, USA). The separation of the 5-μL aliquot was performed on a reverse-phase Acquity UPLC HSS T3 column (2.1 × 100 mm, 1.8 μm particle size, Waters, MA, USA). The eluents were 0.1% formic acid in water (A), and 0.1% formic acid in acetonitrile (B). The gradient was as follows: 0 min, 5% B; 5 min, 10% B; 10 min, 20% B; 12 min, 100% B; 14 min, 100% B; 14.5 min, 10% B; 18 min, 10% B. The flow rate was 0.5 mL min^−1^ and the column temperature was 50°C. Anthocyanins were detected at 520 nm and quantified as petunidin-3-glucoside equivalents using a six-point calibration curve. Furthermore, a validation standard was injected after every tenth injection.

### Transcriptome analysis using RNA-sequencing

Total RNA was extracted using the Spectrum Plant Total RNA kit® (Sigma-Aldrich). Three biological replicates were used. Library construction, sequencing, mapping to reference genome, and quality control were performed at Australian Genome Research Facility (AGRF; www.agrf.org.au) (Illumina Stranded mRNA with 150 paired end reads and sequencing to 20M depth). The primary sequence data were generated using the Illumina DRAGEN BCL Convert 07.021.624.3.10.8 pipeline. Data yield for the 48 samples from the pipeline ranged from 6.91Gb to 12.7 Gb, with mean yield of 9.3 Gb. Methods at AGRF followed the strict requirements of the International standard ISO17025 for quality control, testing and calibration. The per-base sequence quality for the 48 samples was excellent, with >87% bases above Q30 across all samples. Reads were mapped using the STAR aligner (v2.5.3a) to the *Solanum muricatum* genome downloaded from http://songlab.bio2db.com/pepino.html. Mapping QC results are presented in **Supplementary Figure 1**. Counts were normalized using the Relative Log Expression method in the DESeq2 package (Love et al., 2014). Principal component analysis was performed using common variance on the top 500 most variable genes, to check consistency of the biological replicates. The transcriptome response of the three biological replicates was similar, allowing differences in genotype, tissue type and developmental stage to be examined (**Supplementary Figure 2**).

### Phylogenetic analysis

Sequence alignment was performed using Geneious Prime® (version 2022.0.1) Muscle alignment with eight iterations and the phylogenetic tree was constructed using the Neighbor-Joining method with 1000 bootstrap replicates.

### Real time qPCR expression analysis

Total RNA of skin and flesh of the two pepino genotypes, PS and YS, was extracted using the Spectrum Plant Total RNA Kit (Sigma -Aldrich, St. Louis, MO, USA). Removal of genomic DNA and first strand cDNA synthesis was carried out using the QuantiTect Reverse Transcription kit (Qiagen, Hilden, Germany) according to the manufacturer’s instructions.

Real-time qPCR DNA amplification and analysis was carried out using the LightCycler 480 Real-Time PCR System (Roche Diagnostics, Switzerland), with LightCycler 480 software version 1.5. The LightCycler 480 SYBR Green I Master Mix (Roche) was used. Each reaction volume was 10 µL; reactions were run with four replicates and a water control was included in each run. qPCR conditions were as follows: 5 min at 95°C, followed by 40 cycles of 5 s at 95°C, 5 s at 60°C, and 10 s at 72°C, followed by 65–95°C melting curve detection. The qPCR efficiency of each gene was obtained by analyzing the raw fluorescence data using the LinRegPCR (2014.x) software (Ruijter et al., 2009). Used primer sequences are listed in **Supplementary Table 1**. These were normalized to elongation factor-1 of pepino (Sm06G02378).

### Gene cloning and sequence analysis

Sequences of candidate genes were identified by BLAST (Altschul et al., 1997) from the *S. muricatum* pepino genome (Song et al., 2022) and PCR-amplified from leaf cDNA using specific primers and Phusion® High Fidelity DNA polymerase (Thermo Fisher Scientific, USA). PCR products of anthocyanin-related regulatory genes (*SmMYB113* and *SmATV*) and genes that encode enzymes of the anthocyanin pathway (*SmDFR* and *SmF3’5’H*) were cloned into pENTR-TOPO (ThermoFisher) followed by In-Fusion cloning (Takara Bio) into pHEX2. Clones were confirmed by sequencing (Macrogen).

### Transient assays of gene function

Transient assays were performed in tobacco as previously reported (Hellens et al., 2005). Approximately 300 μL of *Agrobacterium tumefaciens* GV3101 culture containing genes of interest were infiltrated into a young *N. tabacum* leaf. Final color development was evaluated by taking digital photographs four days after the infiltration.

### Stable transformation of pepino

*SmMYB113* was transformed into etiolated hypocotyls of pepino (*S. muricatum*) yellow-skinned selection (YS) following a previously described transformation protocol (Horsch et al., 1985) adapted to use hypocotyls as explants. Briefly, etiolated hypocotyls were cut into 5-mm long pieces. Explants were inoculated with 10 mL *Agrobacterium* culture for 10 min and blotted dry with sterile filter paper. Inoculated explants were transferred onto co-cultivation medium for 3 days in the dark. After co-cultivation, explants were transferred onto regeneration medium (MS basal salts and vitamins (Duchefa, Haarlem, The Netherlands), 3% sucrose, 0.7% agar (Invitrogen, Waltham, MA, USA), 1 mg/L BAP + 0.1 mg/L NAA and 300 mg/L Timentin) and selection media (MS basal salts and vitamins (Duchefa), 3% sucrose, 0.7% agar (Germantown, 0.1 mg/L NAA and 300 mg/L Timentin) to induce adventitious shoots from callus tissue. Selection was by the inclusion of 150 mg/L kanamycin in the medium. A single shoot was selected from each explant and transferred onto elongation medium which also included 150 mg/L kanamycin. Three to four weeks later shoots started to develop roots. Once roots were established, rooted plants were transferred into potting mix and moved to glasshouse conditions between 20 to 24°C using natural daylight with extension to 16 h.

## Results

### Anthocyanins are responsible for differences in pepino fruit pigmentation

**Figure 1:**
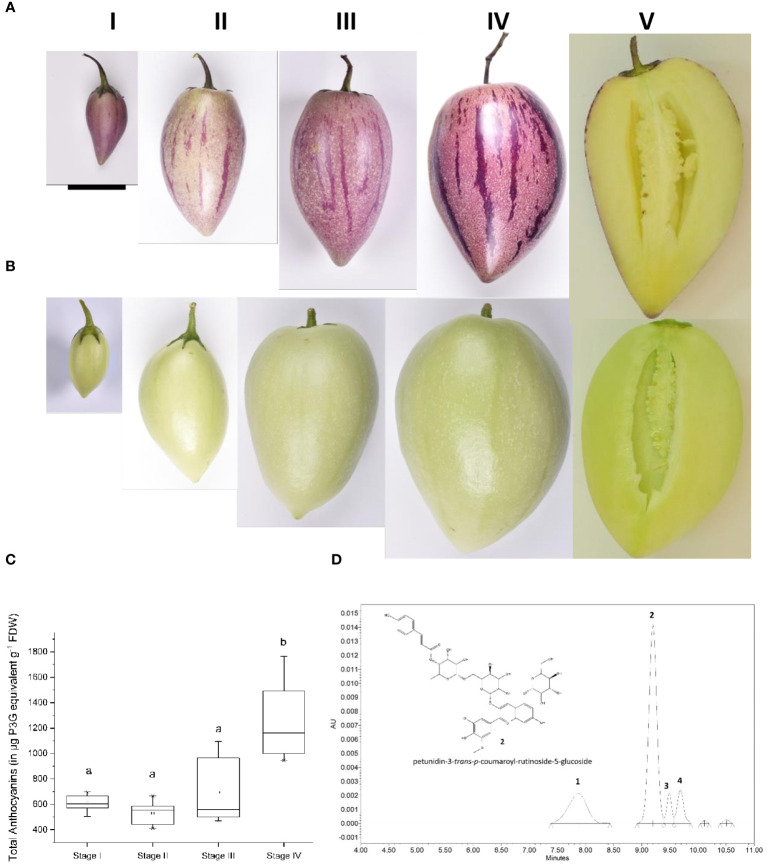
Stages of pepino fruit development. I (20 Days after flowering, DAF), II (34 DAF), III (48 DAF), IV (73 DAF) for the purple-skinned **(A)** selection and the yellow-skinned **(B)** selection. **(C)** Total anthocyanin content in the purple-skinned selection of pepino at four different stages of development (n =9). Data were expressed in µg of petunidin-3-glucoside equivalent (P3G) per gram of freeze-dried weight (FDW). Stages with different letters are significantly different at the *p <*0.05 level. Anthocyanins are absent in the yellow-skinned cultivar. **(D)** UPLC-PDA chromatogram of pepino skin (Stage IV) recorded at 520 nm, with molecular structure of the predominant anthocyanin 2 identified as petunidin-3-*trans*-*p*-coumaroyl-rutinoside-5-glucoside. Scale bar on **(A)** is equivalent to 4 cm.

Two pepino selections that differ in anthocyanin pigmentation of the fruit skin, denoted purple skin (PS) or yellow skin (YS), were chosen for analysis. They produce fruit of comparable size, shape, growth rate and yellow flesh color (**Figures 1A, B**). Anthocyanins were absent from the skin and flesh of the YS genotype, throughout fruit development. Both YS and PS genotypes can produce anthocyanins in leaves and petals, although PS has much darker purple foliage and petals (**Supplementary Figure 3**). Total anthocyanin concentrations were evaluated throughout the fruit development in the PS genotype (**Figure 1C**). The total amount of anthocyanin was significantly higher at stage 4 than in stages 1 to 3, reaching 1.7 mg g^-1^ (dry weight or DW) at stage 4.

Anthocyanin composition was first determined in the skin of the PS genotype by Ultra Performance Liquid Chromatography-High-Resolution Mass Spectrometry (UPLC-HR/MS) and Ultra Performance Liquid Chromatography- diode Array Detector (UPLC-DAD) and compared with available data from the literature. Four predominant anthocyanin compounds were detected in the profiles (**Figure 1D**). Peak 2 represented about 60% of total anthocyanins and was identified as petunidin-3-*p*-coumaroyl-rutinoside-5-glucoside (**Figure 1D**; **Supplementary Figure 4**). The presence of this anthocyanin has previously been reported in other fruits of the Solanaceae family, such as purple tomatoes (*Solanum lycopersicum* L.) (Silva Souza et al., 2020), and purple-fleshed potato tubers (*Solanum tuberosum* L.) (Andre et al., 2007). When anthocyanins are acylated, both cis- and trans- isomers can be found (Saha et al., 2020). Considering the predominance of trans isomers in *Solanum* species, it is likely that peak 2 is petunidin-3-trans-coumaroyl-rutinoside-5-glucoside (also known as petanin), whereas peak 3 is its cis- stereoisomer.

The other anthocyanin compounds followed the same substitution pattern: -3-acylrutinoside-5-glucoside and exhibited a similar anthocyanidin fragment at m/z 317, indicative of petunidin derivatives. Consequently, peaks 1 and 4 were identified as petunidin-3-caffeoyl-rutinoside-5-glucoside and petunidin-3-feruloyl-rutinoside-5-glucoside, respectively.

There were no significant differences in the proportions of the different anthocyanins at the four developmental stages investigated, suggesting that there is a common regulatory system for all four anthocyanin compounds.

### RNAseq analysis of the anthocyanin biosynthetic and regulator genes

To study the transcripts associated with anthocyanin accumulation in pepino, skin and flesh samples were collected from PS and YS genotypes over the four developmental stages and subjected to RNA-seq. A comparative analysis between purple and yellow tissues at the same developmental stage was performed and differentially expressed genes (DEGs) with the minimum of log_2_-fold change in expression (DESeq, adjusted *p*-value<0.05, mean count 100) between the purple and yellow cultivars were identified. This analysis revealed 1540 up-regulated DEGs and 1467 down-regulated DEGs in skin, and 1524 up-regulated DEGs and 1269 down-regulated DEGs in flesh, when PS was compared with YS. Of these genes, there was an enrichment for candidate genes related to anthocyanin biosynthesis or regulation of this pathway.

**Figure 2:**
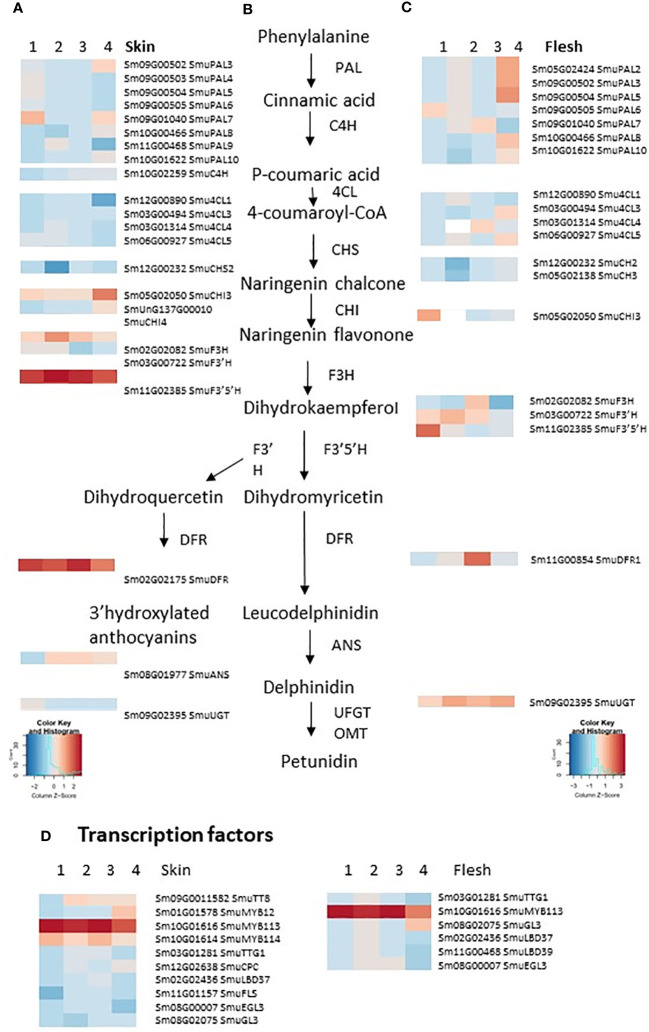
Heatmaps showing the differential expression of anthocyanin biosynthesis, regulatory and transport genes in the purple-skinned selection vs yellow-skinned selection pepino skin **(A)** and flesh **(C)**, in four stages of development. An abbreviated version of the anthocyanin biosynthetic pathway is provided **(B)**. **(D)** Transcription factors implicated in regulating this pathway. The color of any given cell represents the normalized read values, with red representing high expression levels and blue representing low expression levels, with two color keys separate for skin and flesh. Genes are annotated according to Song et al. (2022).

Previously, 17 pepino regulatory genes and 11 biosynthetic enzymes have been identified as participating in anthocyanin biosynthesis (Song et al., 2022). Most of these gene models were differentially expressed between YS and PS (**Figure 2**). In our study, a total of 25 anthocyanin-related DEGs were consistently up-regulated, with four down-regulated in PS skin samples compared with YS (**Figure 2**). Four genes were DEGs but had variable expression across skin developmental stages. In flesh, 13 and seven DEGs were consistently up- or down-regulated, respectively and when comparing the DEGs in skin and flesh, eight biosynthetic genes were up-regulated (*SmuPAL6, SmuPAL7, SmuPAL10, Smu4CL3, Smu4CL4, SmuCHI3, SmuF3’H and SmuUGT*). Also, the anthocyanin-related R2R3 MYB regulator *SmuMYB113* was up-regulated in both PS tissues, whereas the bHLH SmuTT8 was up-regulated in skin only. In contrast, *Smu4CL1* and *SmuCHS2* (both anthocyanin biosynthesis-related genes) were down-regulated in both skin and flesh tissues. In addition, five genes were DEGs but had variable expression across flesh developmental stages.

**Figure 3:**
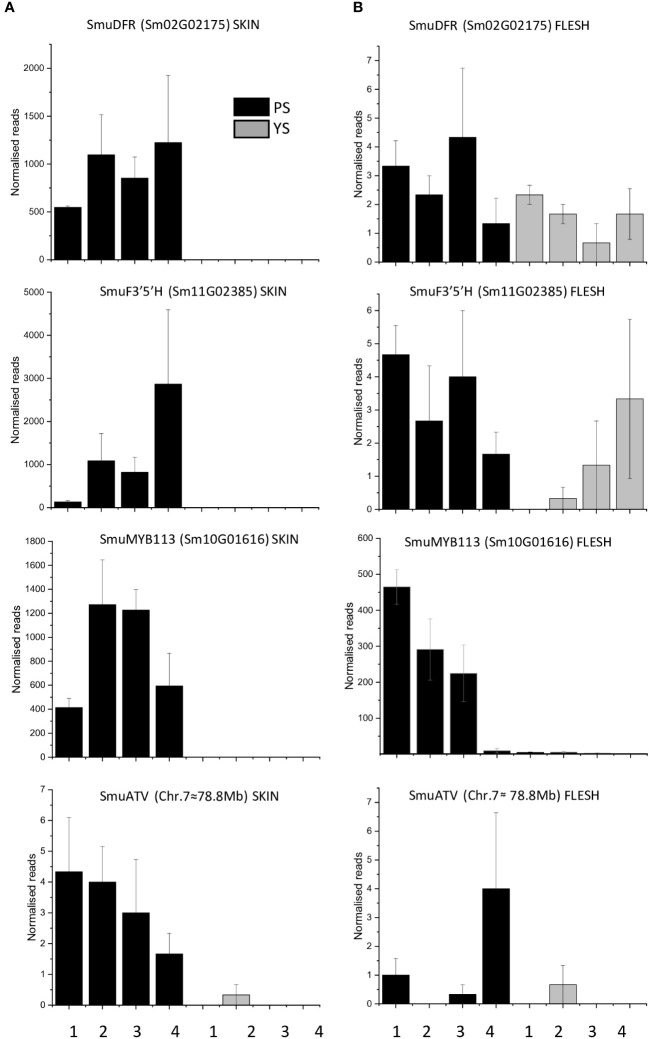
Transcript levels in skin **(A)** and flesh **(B)** of anthocyanins biosynthetic genes (SmuDFR and SmuF3’5’H) and regulatory genes (SmuMYB113 and SmuATV) in the skin and flesh of two pepino selections at four developmental stages. Error bars represent standard errors of the means (STE) of three biological replicates.

Transcript reads of *SmuDFR2* (Sm02G02175) were significantly higher in the purple-skinned genotype than in the yellow-skinned cultivar (**Figure 3**). Reads of less than 10 per stage were present in flesh of both PS and YS (**Figures 3A, B**). A similar trend was seen with SmuF3’5’H (Sm11G02385), where transcripts were significantly higher in the PS genotype than in the YS cultivar. Reads peaked at stage 4, which correlated with the highest concentrations of anthocyanins (**Figure 1**). In the flesh, reads for SmuF3’5’H were again less than 5 normalized reads per stage.

An examination of candidate transcriptional regulators of anthocyanin-related enzymes was made. There were four candidate MYB transcription factors in the DEG lists, as well as a bHLH, which is homologous (69% identity) to AtTT8 (**Figure 2A**) and was previously annotated as SmuTT8 (Song et al., 2022) (**Table 1**). This *TT8*-like bHLH was highly expressed in PS skin, but not as well expressed in PS flesh. However, the R2R3 MYB *SmMYB113* was consistently up-regulated, in both skin and flesh of the purple-skinned selection. Transcript counts were high in all purple fruit tissues (**Figure 3B**). In contrast, reads were absent or very low in YS fruit tissues.

**Table 1:** Normalized reads of candidate transcription factors in pepino selections.

gene model	Description	PS skin	YS skin	PS flesh	YS flesh	Fold change Up in PS vs YS^a^	
		Total normalized reads across all stages				Skin	Flesh
Sm12G00739	SmuMYB11	0	0	0	0		
Sm01G01578	SmuMYB12	95	97	0	0		
Sm06G02794	SmuMYB111	0	0	0	0		
Sm10G01616	SmuMYB113	3511	0	998	12	11.6	6.0
Sm10G01614	SmuMYB114	76	1	22	0		
Sm10G01615	SmuPAP1	0	0	0	0		
Sm01G00097	SmuPAP2	0	0	3	0		
Sm09G01582	SmuTT8/bHLH	1099	93	47	10	2.8	
Sm08G02075	SmuGL3/bHLH	1350	1145	1036	888		
Sm08G00007	SmuEGL3/bHLH	3535	4149	4297	4679		
Sm03G01281	SmuTTG1/WD40	37	40	40	35		
Sm05G00345	SmuMYBL2	33	0	0	0		
Sm12G02638	SmuCPC	489	256	508	345	1.6	
SmATV	SmuATV	13	0	6	1		
Sm02G02436	SmuLBD37	956	729	94	58		
Sm01G03283	SmuLBD38	1823	1795	1596	1713		
Sm11G00468	SmuLBD39	554	307	844	823		

^a^Data presented only if fold change ≥2, p value ≥0.05 and mean count over samples ≥50. PS, purple-skinned; YS, yellow-skinned.

Candidate repressors of anthocyanins, such as R3 MYBs and LOB domain TFs, did not explain the phenotypes, as they were not up-regulated either in YS, or in PS flesh tissues (**Figure 2**). In addition, a homologue of the tomato anthocyanin repressor *SlATV*, which we termed *SmATV*, was identified by blast match in the pepino genome (chr.7, 78.8Mb). Reads mapping to this gene model (previously un-annotated) were low, but present in both skin and flesh (**Figures 3A, B**). Furthermore, a flavonol-related subgroup 7 MYB (*SmuMYB12*) was expressed in skin of both selections (**Table 1**), and therefore increased flux of precursor substrates away from anthocyanins does not appear be the cause of the YS phenotype.

### Phylogenetic analysis of *SmuMYB113, SmuATV* and MYB repressors of the anthocyanin pathway

**Figure 4:**
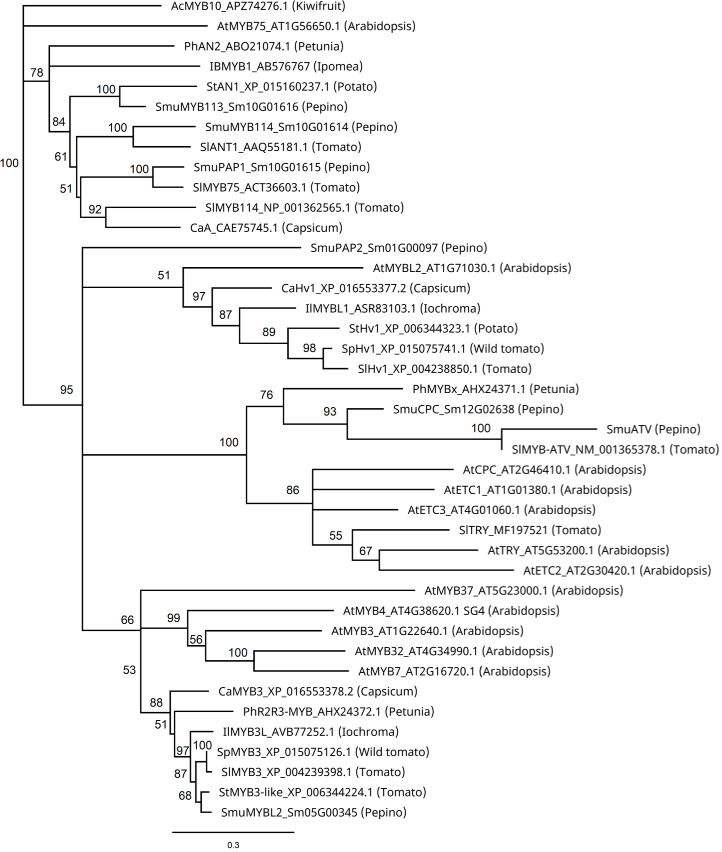
Phylogenetic relationship analysis of pepino MYBs and known anthocyanin MYB regulators from other species. Sequences were aligned using predicted protein sequences in Geneious (version Geneious Prime^®^ 2022.0.1) Muscle alignment with eight iterations, and the phylogenetic tree was constructed using the Neighbor-Joining method with 1000 bootstrap replicates.

Phylogenetic analysis of these MYBs revealed close similarities between SmuMYB113 and StAN1 of potato, and SmuMYB114, and SlANT1 of tomato (**Figure 4**). In potato, StAN1 is a key regulator of skin coloration (Payyavula et al., 2013). Although differentially expressed, SmMYB114 showed low expression in pepino (**Table 1**). Therefore, both expression and sequence similarity suggest SmuMYB113 is the causative controller of pepino skin anthocyanin content. However, this does not explain the flesh phenotype, where high levels of SmMYB113 expression are seen, yet no anthocyanins accumulate. In this phylogenetic tree, *SmuATV* clustered with a group of R3 MYB TFs. Within this group, *SmuATV* was closely related to *PhMYBx* and *SlMYBATV*, both negative regulators of anthocyanin biosynthesis (Kroon, 2004; Albert et al., 2011; Albert et al., 2014b).

### qPCR analysis validation of candidate genes

**Figure 5:**
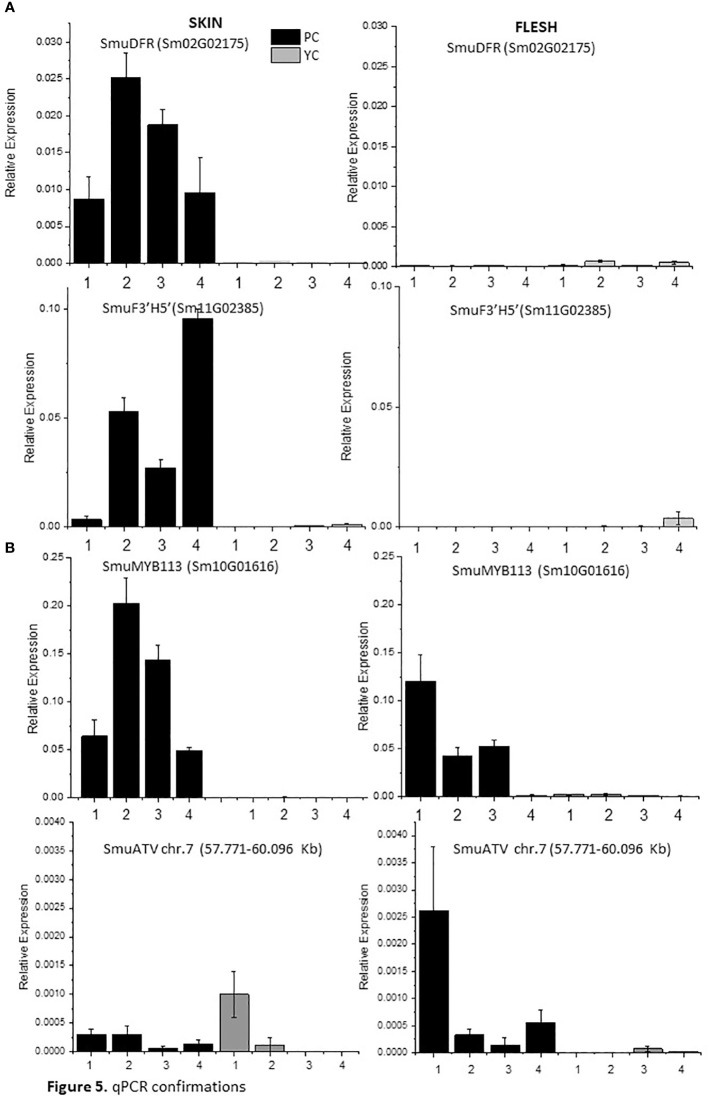
Expression analysis of anthocyanin biosynthetic genes SmuDFR and SmuF3’5’H and anthocyanin regulatory genes SmuMYB113 and SmuATV, across four developmental stages of two pepino selections. **(A)** Relative expression levels of biosynthetic genes SmuDFR and SmuF3’5’H and regulatory genes, and **(B)** regulatory genes SmuMYB113 and SmuATV in purple-skinned and yellow-skinned selection skins.

Expression of anthocyanin-related genes was verified using RT-qPCR. Accumulation of transcripts of anthocyanin biosynthetic genes *SmuDFR2* and *SmuF3’5’H* was confirmed in purple skin, but these were absent from yellow skin and flesh at all studied stages of fruit development (**Figure 5**). An increase at 34 DAF for *SmuDFR* was followed by a decline at later fruit developmental stages, while transcripts of *SmuF3’5’H* increased at 34 DAF and peaked at 73 DAF.

The expression of *SmuMYB113* and *SmuATV* in flesh and skin was examined using qPCR. For *SmuMYB113*, the purple-skinned stage 2 of development showed the highest value, followed by a decline. For yellow-skinned (YS) pepino, the values were significantly lower in all stages of development, in both skin and flesh. However, in the flesh of the purple-skinned genotype, high levels of expression were seen at three of the four stages. This confirms patterns seen with RNAseq, and suggests a repressive mechanism exists in the pepino flesh. Expression levels of the potential repressor *SmuATV* were low in the purple- and yellow-skinned cultivars. However, PS had higher levels of expression generally than YS.

As *SmuMYB113* appears to be a key regulatory difference between the purple-skinned and yellow-skinned cultivars, further correlations were made between its expression and other putative target genes within the phenylpropanoid and flavonoid pathways. Early steps in the anthocyanin pathway, such as *PAL, C4H, 4CL, CHS*, and *F3’H*, were all well expressed in yellow skin and flesh tissues of both selections (**Table 2**). This is despite the fact that no anthocyanin accumulated in yellow skin or flesh, or in the flesh of the PS selection. In skin tissues, there was a strong positive correlation between *SmuMYB113*/anthocyanin and expression of *CHI3*, *F3’5’H, DFR*, and the *GST*, *TT19*. An exception was the previously annotated UFGT (Sm09G02395) (Song et al., 2022), which showed high expression in all fruit tissues. By blasting the Arabidopsis anthocyanidin 3-O-glucosyltransferase (At5g17050) and grape UFGT (Ford et al., 1998), better matching pepino gene models were retrieved, such as Sm10G01884 (**Table 2**), which was expressed in a skin-specific manner, especially in PS tissue. We therefore hypothesize that the absence of *SmuMYB113* in the yellow-skinned selection of pepino results in a loss of expression of later steps in the anthocyanin pathway. This would also lead to a loss of expression of the bHLH partner, *SmTT8*, which is known to be part of a hierarchy of regulation in solanaceous plants (Montefiori et al., 2015). However, this does not explain the presence of *SmuMYB113* expression in the flesh of the purple-skinned selection, where there is no elevation of expression of these later biosynthetic steps, or indeed of the co-regulator *SmTT8*.

**Table 2:** Normalized reads of candidate biosynthetic-related genes in pepino selections.

gene model	Description	PS skin	YS skin	PS flesh	YS flesh	Fold change Up in PS vs YS^a^	
		Normalized reads across all stages				Skin	Flesh
Sm10G01622	SmuPAL10	9025	4105	11067	2420	1.5	1.1
Sm09G00502	SmuPAL3	11584	5056	7332	7129	1.6	
Sm09G00503	SmuPAL4	8246	4366	1818	2460		
Sm09G00504	SmuPAL5	8475	5022	2159	3176		
Sm09G00505	SmuPAL6	14	10	12	3		
Sm10G02259	SmuC4H	8794	4433	1957	1338		
Sm03G00494	Smu4CL3	2244	1352	725	444		
Sm03G01314	Smu4CL4	673	416	37	32		
Sm06G00927	Smu4CL5	6096	2973	4379	4094		
Sm05G02050	SmuCHI3	328	12	18	3		
SmUnG137G00010	SmuCHI4	1600	1249	1232	1420	1.1	
Sm05G02138	SmuCHS	30706	26715	33015	56022		
Sm02G02082	SmuF3H	7273	235	126	417	4.9	
Sm03G00722	SmuF3’H	874	631	665	329		0.9
Sm11G02385	SmuF3’5’H	4870	1	13	5	11.0	
Sm02G02175	SmuDFR	7846	3	20	3	11.1	
Sm08G01977	SmuANS	2644	1	17	4	10.2	
Sm09G02395	SmuUFGT	514	183	1086	129	1.1	2.9
Sm10G01884	SmuUFGT-like	4011	190	1	0	4.8	
Sm02G01868	SmuTT19	5962	6	5	1	10.2	

^a^Data presented only if fold change ≥0.5, p value ≥0.05 and mean count over samples ≥ 50. PS, purple-skinned; YS, yellow-skinned.

### Functional assays of SmuMYB113 and SmuATV in tobacco

**Figure 6:**
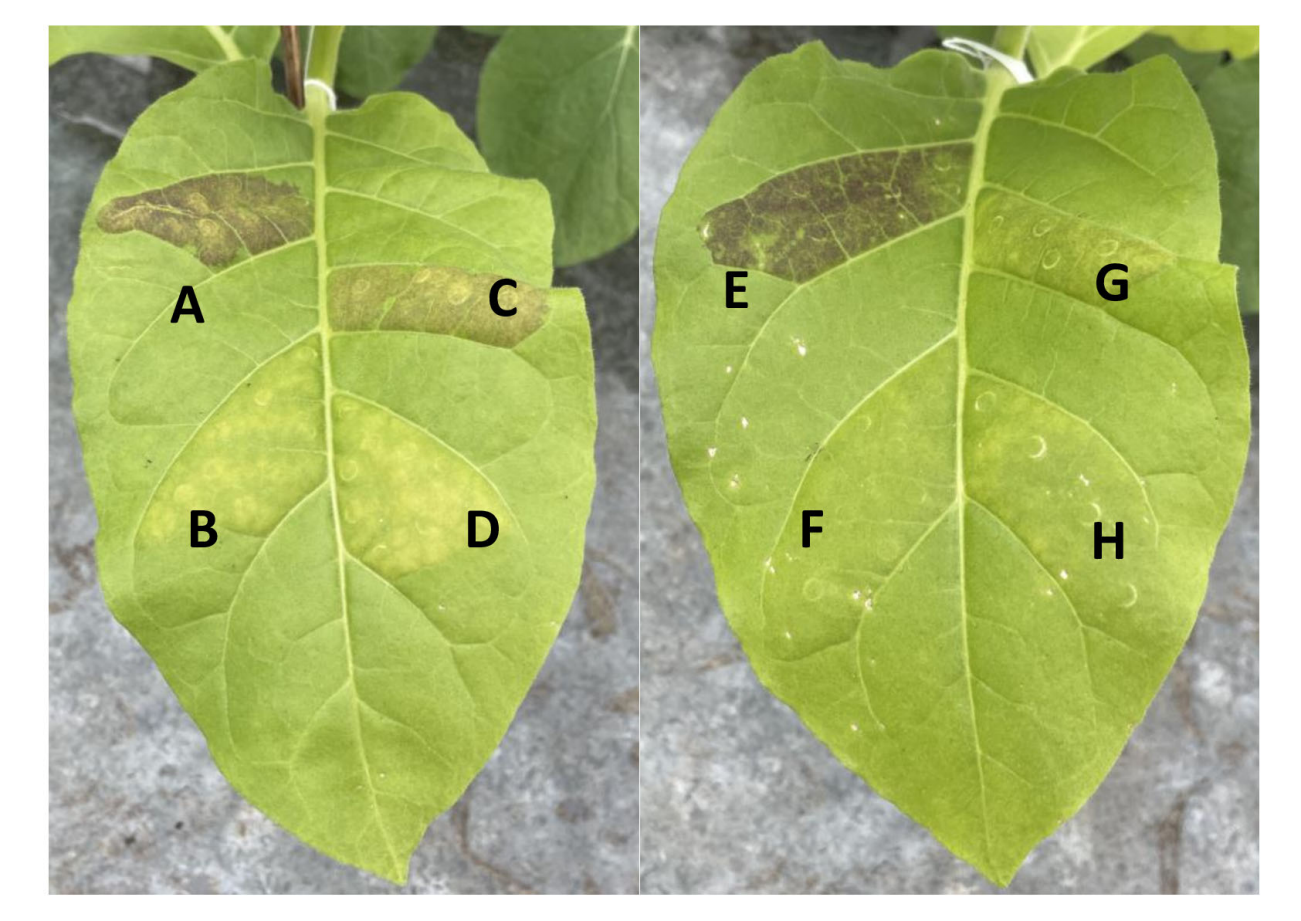
SmuMYB113 and SmuATV regulate anthocyanin accumulation in *Nicotiana tabacum* leaves. *N. tabacum* tobacco leaves were infiltrated with *Agrobacterium* strains expressing anthocyanin activators *StAN1* and *SmuMYB113*, or the R3-MYB repressor *SmuATV* is various combinations. *Agrobacterium* carrying a plasmid with GUS-only was included as a negative control and to maintain equivalent proportions of each strain. Anthocyanin accumulation (visibly assessed) 4 d post-infiltration. **(A)** StAN1 + GUS, **(B)** GUS only **(C)** StAN1 + SmuATV **(D)** SmuATV with GUS, **(E)** SmuMYB113 + GUS, **(F)** GUS only **(G)**. SmuMYB113 + SmuATV and **(H)**. SmuATV (+ GUS).

To functionally test the R2R3 MYB SmuMYB113 and R3 MYB SmuATV, their full-length coding regions were cloned, and placed under the control of the *35S* promoter. These sequences were infiltrated using *Agrobacterium* into tobacco leaves (Espley et al., 2007). Infiltration of *SmuMYB113* activated anthocyanin production in tobacco leaves (**Figure 6**). No anthocyanin production was observed with the negative control GUS construct. The potato *StAN1* (NCBI protein accession number: AKA95392) construct (Liu et al., 2016) was infiltrated as a positive control and resulted in strong activation of anthocyanin accumulation (**Figure 6**). In contrast, co-infiltration of *StAN1* with *SmuATV* significantly reduced anthocyanin accumulation compared with StAN1 alone. Similarly, when *SmuATV* was co-infiltrated with *SmMYB113*, there was no anthocyanin accumulation, suggesting strong repression of the R2R3 MYB by this R3 MYB. These findings support the hypothesis that *SmuATV* acts as a repressor of anthocyanin accumulation.

### Functional characterization of SmMYB113 in transformed pepino lines

**Figure 7:**
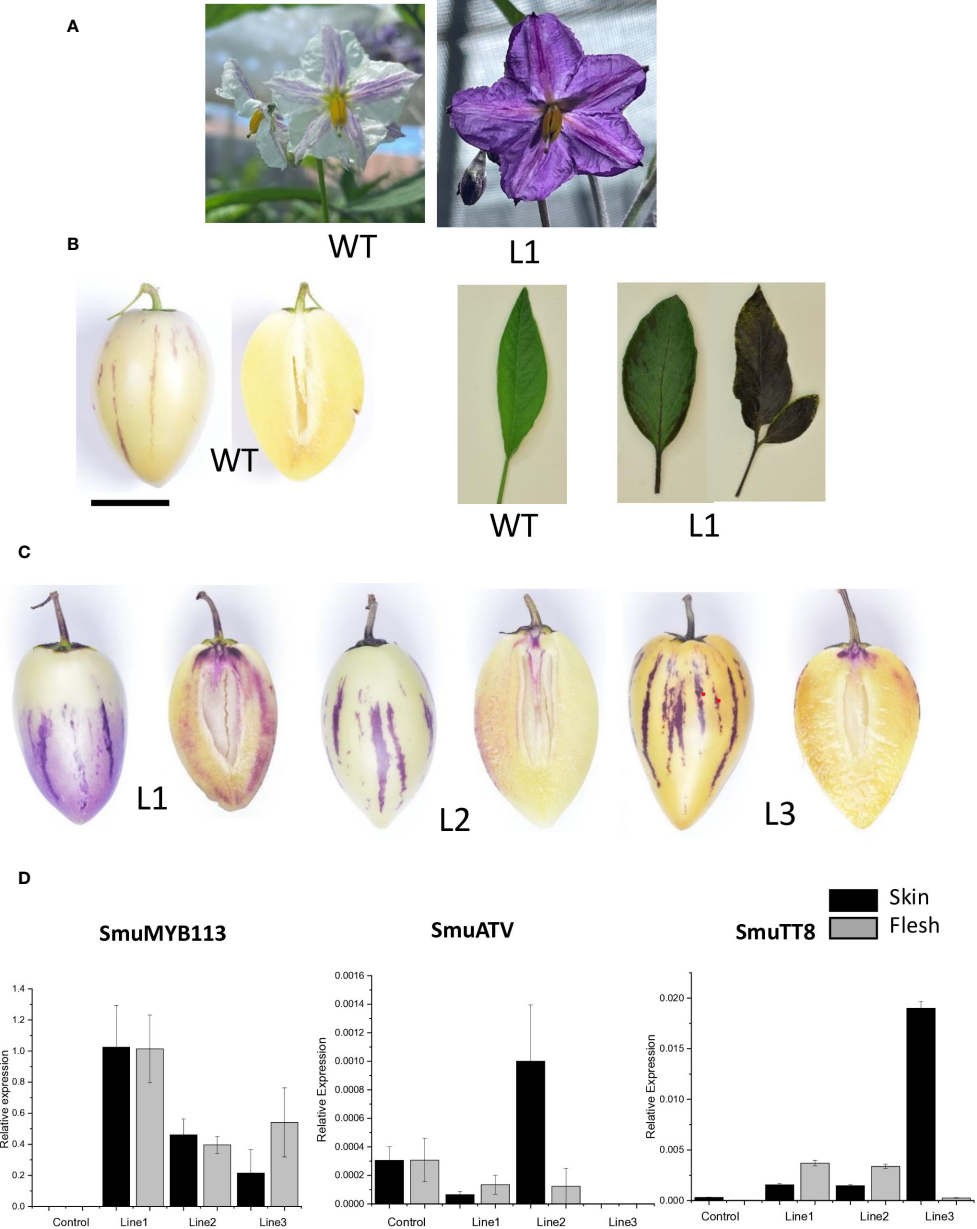
Overexpression of SmuMYB113 induced anthocyanin accumulation in flowers, leaves and fruit skin and flesh in pepino plants. **(A)** Wild-type (WT) flower phenotype and line overexpressing SmuMYB113 phenotype (L1). **(B)** Left: WT pepino fruit phenotype. Right: WT leaf phenotype and line over-expressing SmuMYB113 phenotype (L1). **(C)** Transformed lines (L1, L2 and L3) showing anthocyanin accumulation in skin and flesh. **(D)** Transcript levels in skin and flesh of anthocyanin-regulatory genes SmuMYB113 and SmuATV of three independent lines overexpressing SmuMYB113.

To characterize the effect of SmMYB113 on pepino anthocyanin biosynthesis, we transformed pepino (YS genotype) with a 35S:*SmMYB113* overexpression construct. Three independent lines were produced that showed intense purple pigmentation in leaves and flowers (**Figures 7A, B**). Fruit developed normally and had visibly enhanced purple color (**Figure 7C**). These fruits were tested by qPCR, which showed increased expression of the *SmuMYB113* gene in both fruit skin and flesh (**Figure 7D**). A correlation between transgene expression and fruit skin and flesh color intensity was apparent, with transgenic pepino Line 1 showing the highest *SmuMYB113* transcript levels and the darkest skin and flesh (**Figures 7C, D**). In contrast, *SmuATV* transcript was higher in the skin and flesh of a control fruit than in Lines 1 and 3, although increased *SmuATV* was detected in the skin of Line 2. Transcript abundance of *SmuTT8* was also elevated in the transgenic lines, compared with the untransformed YS control. In the flesh of YS wild-type lines, there were very few reads of TT8 in the RNA seq dataset (**Table 1**). However, when MYB113 was overexpressed as a cDNA transgene, TT8 transcript was induced. Over-expression of *SmuMYB113* therefore changes a YS phenotype into a plant with purple-skinned fruit, although the flesh color was still not as intense as expected from a strong promoter. This is further evidence that a strong repressive background is not fully overcome by over-expression of the R2R3 MYB.

## Discussion

Enhancing fruit quality, particularly by providing higher vitamin and antioxidant contents, can provide numerous health benefits. For this reason, accumulation of compounds such as anthocyanins have been studied in many plants. Anthocyanin biosynthesis has been widely characterized in solanaceous species (Albert et al., 2011; Kiferle et al., 2015; Montefiori et al., 2015; Liu et al., 2016; Sun et al., 2020; Yan et al., 2020; Zhou et al., 2022). In tomato, R2R3 MYB genes *SlANT1, SlANT1like* and *SlAN2* have been identified as positive anthocyanin regulators (Kiferle et al., 2015; Colanero et al., 2020). Also in tomato, the R3 MYB gene, *SlATV*, has been shown to be a negative regulator for anthocyanin biosynthesis (Yan et al., 2020). In pepino, key anthocyanin-related genes have been reported (Song et al., 2022), as well as several potential transcription factors regulating the anthocyanin pathway, but these are yet to be characterized.

In this current study we characterized two candidate genes: *SmuMYB113*, encoding an R2R3-MYB TF, and *SmuATV*, encoding an R3-MYB TF. Our results suggest that expression of *SmuMYB113* is the key difference between the two evaluated pepino selections, PS and YS. This is based on RNA-seq and qPCR data, as well as tests of gene function in both tobacco and pepino transgenics. A previously un-annotated MYB, *SmuATV*, was shown to be a strong repressor of anthocyanin accumulation, so this could be a candidate for inhibiting accumulation of anthocyanins in the pepino flesh.

Although expression of *MYB113* appears to be a key difference between purple-skinned and yellow-skinned pepinos, there is still expression in flesh of purple-skinned cultivars where no anthocyanins accumulate. In the *35S*:*SmuMYB113* transformants, which is a yellow-skinned selection, flesh expression of the transgene was high but once again flesh anthocyanins did not accumulate to a high concentration. In the close relative, eggplant (*Solanum melongena*), it has been reported that eggplant *MYB113* significantly increased the anthocyanin and flavonol content in both peel and fruit pulp (Yang et al., 2022).

The RNAseq dataset revealed a large number of differentially expressed transcripts, obtained from comparing the tissues of pepino skin and flesh (PS vs PY) at four stages of development. Genes encoding early anthocyanin biosynthetic enzymes (*PAL, C4H, 4CL, CHI* and *CHS*) were well expressed in fruit tissues even in YS, and in the flesh of PS (**Table 2**). However, the late stages of the anthocyanin pathway (*F3H, F3’5’H, DFR, ANS, GST*) were low in expression in tissues with low anthocyanin content. The transcript levels of *SmuF3’5’H* and S*muDFR* were validated using qPCR and were significantly up regulated in PS skin, compared with PS flesh, and with YS skin and flesh. These results align with the observed distribution pattern of anthocyanins in both the skin and flesh of the pepino fruit (**Figure 1C**). The exception to this is the expression of *UFGT* (Sm09G02395), which showed high expression levels in yellow and purple tissues (**Table 2**). However, re-examination of the pepino genome suggested better candidate genes for *UFGT* (Sm10G01884) that did corelate with anthocyanin contents (**Table 2**). Recently transcriptomic and metabolomic analysis of ramie (*Boehmeria nivea*) was also used to identify UFGT as a key gene in foliage anthocyanin production (Feng et al., 2021).

Candidate regulatory genes were examined in the RNAseq datasets. The transcript of *SmuMYB113* was upregulated in PS skin and flesh in the four stages of development. Its potential partner, *SmuTT8*, was expressed in PS skin, but not highly expressed in flesh. Strong over-expression of *SmuMYB113* activates more expression of *SmuTT8* in transgenics and triggers some activation of the anthocyanin pathway in flesh. We observed no increase in expression of candidate repressors that could explain the lack of activation of *SmuMYB113* in YS, or the lack of anthocyanins in PS flesh. Cao et al. (2017), suggested that in tomato, *SlATV* inhibits the expression of the genes responsible for anthocyanin biosynthesis, leading to a repression in anthocyanin production. Transcript analysis from the pepino RNA-seq data and RT-qPCR, showed that *SmuATV* was expressed in PS skin and flesh. Although *SmuATV* plays a repressive role in regulating the activity of SmMYB113 from functional assays (**Figure 6**), its expression did not correlate with loss of anthocyanins in pepino fruit.

Over-expression of *SmMYB113* in the yellow-skinned pepino selection complemented the phenotype, producing a purple foliage, petals and fruit skin. However, flesh anthocyanin concentrations were still low. This further suggests a strong repressive background in the pepino flesh, despite good expression of the SmuMYB113 transgene. In apple, Espley et al. (2007) identified the MYB component, MdMYB10, as the principal factor for strong flesh pigment accumulation in the MBW complex. However, in solanaceous plants this does not appear to occur. Albert et al. (2014a) showed that petunia MYB AN1-like genes are normally regulated by the MBW complex, as part of a feed-forward mechanism, while Montefiori et al. (2015) identified that a hierarchy of bHLHs is needed for the tobacco MYB activators to bind promoters of the anthocyanin pathway. In YS there is no expression of TT8 in the flesh. However, in lines overexpressing MYB113, some TT8 transcript was induced. Interestingly, skin pigmentation was striped in both purple-fruited cultivars and in transgenics over-expressing *SmuMYB113*. Other levels of regulation, such as degradation of transcripts by RNA processing, or rapid turnover of MYB protein, may well explain why pepino flesh remains relatively un-pigmented or why uneven pigmentation occurs in the skin. If these conundrums can be addressed, considerable improvement in total fruit anthocyanins will be achievable, which is a trait useful in many fruits and vegetables.

## Data availability statement

The RNAseq data presented in this study is deposited in the NCBI repository, BioProject PRJNA1122205.

## Author contributions

MM-S: Conceptualization, Data curation, Formal analysis, Investigation, Methodology, Project administration, Resources, Software, Validation, Visualization, Writing – original draft, Writing – review & editing. DH: Data curation, Formal Analysis, Software, Writing – original draft, Writing – review & editing. AS: Data curation, Formal analysis, Investigation, Software, Writing – original draft, Writing – review & editing. CA: Data curation, Formal analysis, Investigation, Methodology, Validation, Writing – original draft, Writing – review & editing. EV-G: Formal analysis, Investigation, Methodology, Project administration, Resources, Supervision, Writing – original draft, Writing – review & editing. GC: Methodology, Project administration, Resources, Writing – original draft, Writing – review & editing. EB: Conceptualization, Investigation, Methodology, Writing – original draft, Writing – review & editing. AA: Conceptualization, Formal analysis, Funding acquisition, Investigation, Methodology, Project administration, Resources, Supervision, Writing – original draft, Writing – review & editing.

## Supplementary material

Supplementary Figure 2 Principal component analysis using common variance on the top 500 most variable genes in pepino.
