# Supplementary material for: SmuMYB113 is the determinant of fruit color in pepino (Solanum muricatum)
Source: Front Plant Sci. 2024 Jun 20;15:1408202. doi: 10.3389/fpls.2024.1408202 (PMC11222579; doi:10.3389/fpls.2024.1408202)
Supplement: Supplementary Figure 2 — Principal component analysis using common variance on the top 500 most variable genes in pepino. [file DataSheet_1.pdf]

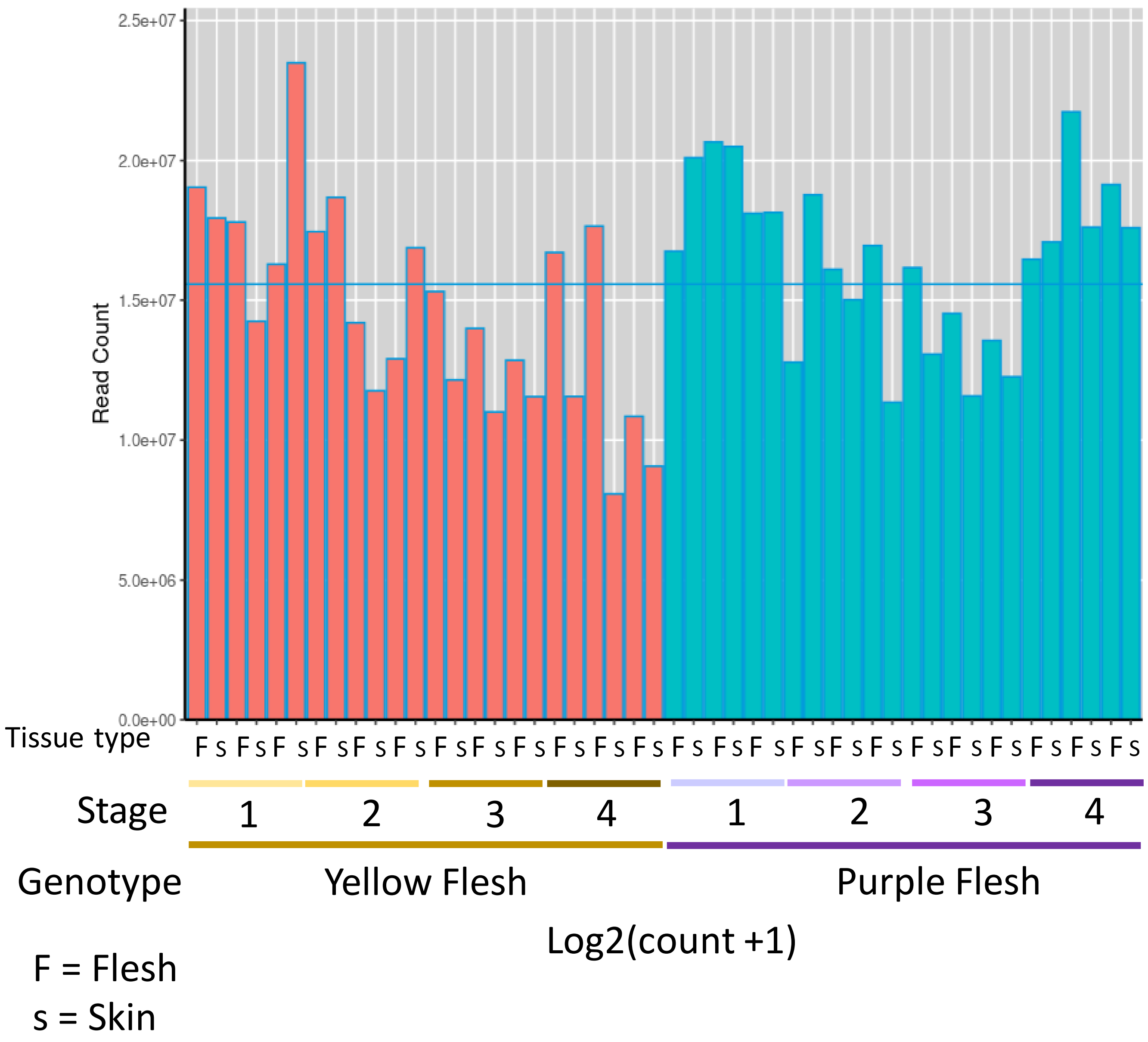

Supplemental Figure 1. Mapping QC results

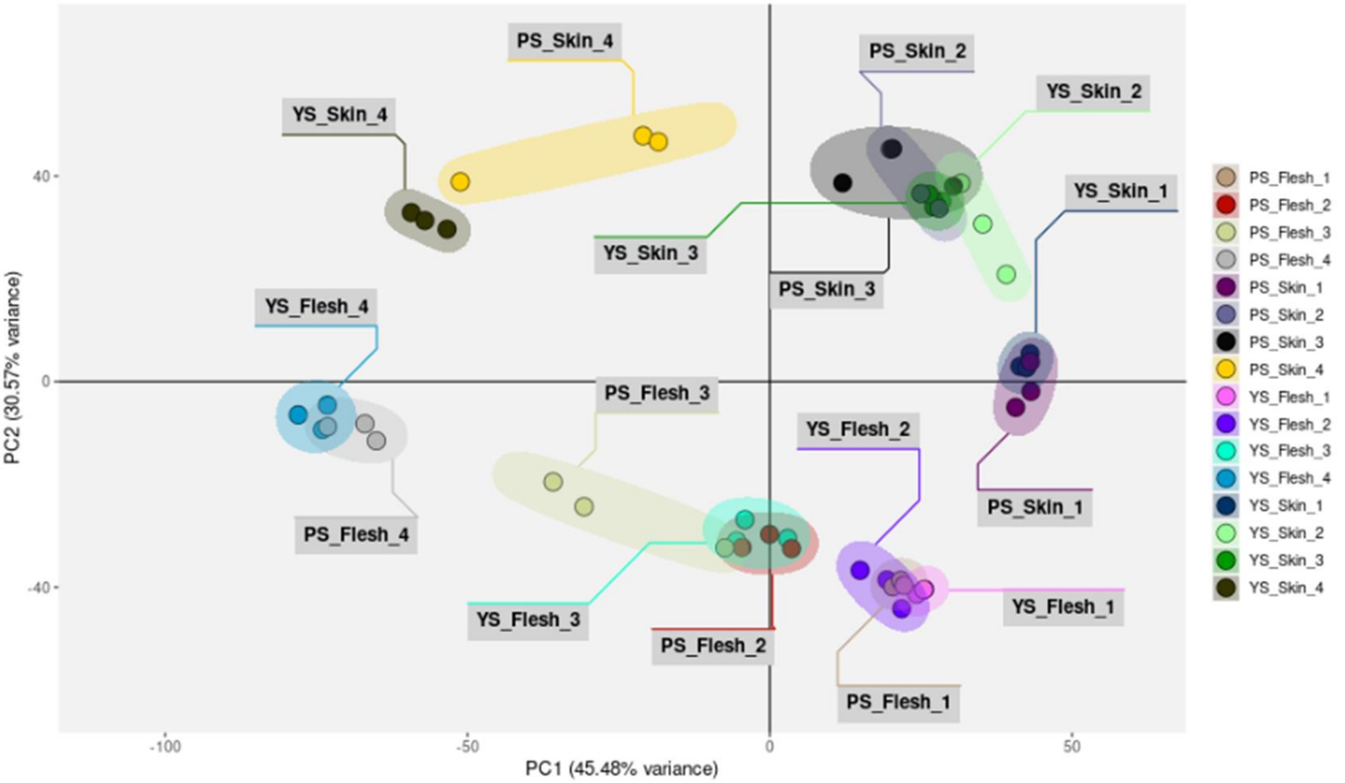

Supplemental Figure 2.

A B

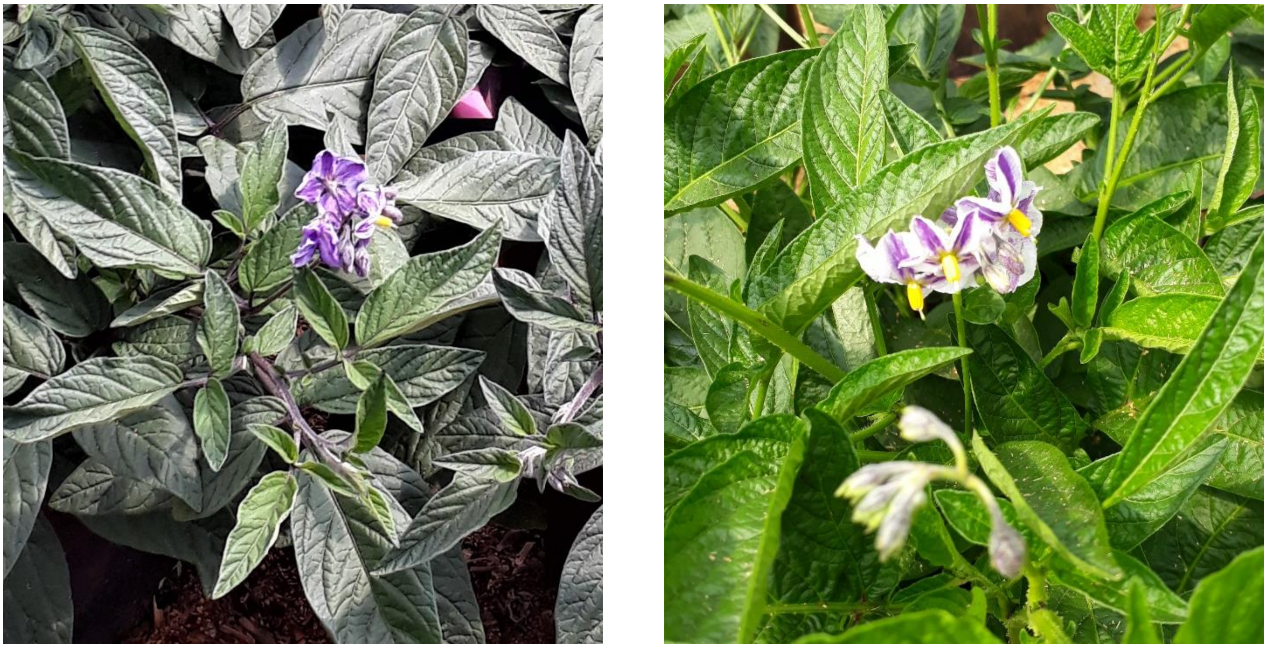

Supplemental Figure 3: Representative pepino leaves and flowers for two pepino selections A. Purple selection (PS) and B. Yellow selection (YS).

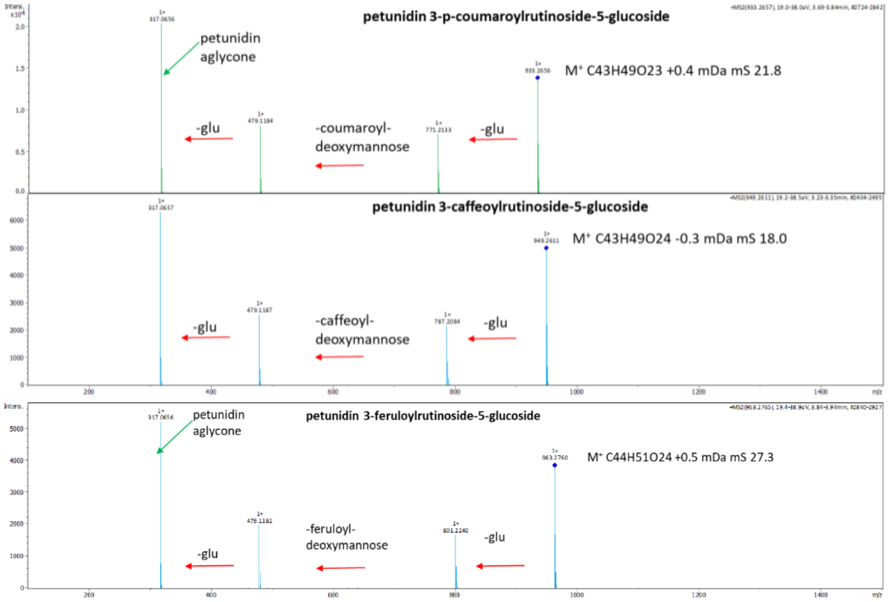

Supplemental Figure 4: High resolution mass spectra and identification of the three predominant anthocyanins: (1) petunidin-3-p-coumaroylrutinoside-5-glucoside; (2) petunidin-3-p-caffeoylrutinoside-5-glucoside; (3) petunidin-3-p-feruloylrutinoside-5-glucoside

Genes	Gene ID	Primer	Primer sequence
**SmDFR**	Sm02G02175	SmDFR-F	TTGGACATCAAGAGTTCCAG
		SmDFR- R	AACCAACAGTTAAGGGAATG
**SmF3’5’H**	Sm11G02385	SmF3’5’H- F	TTCTTCGTCCTGCACCAAAT
		SmF3’5’H- R	GACCCTGAAGTGTGGGAGAA
**SmMYB113**	Sm10G01616	SmMYB113 -F	GTATTGTAAATGAAGAAGCTTCGAAAG
		SmMYB113-R	CCTTAGAGTTGATATTCTCACATGAAGG
**SmATV**	chr.7 (57.771-60.096 Kb)	SmATV-F	CACCTGATCCAACCAGAGTGG
		SmATV-R	ACCTCTCTCTGACCAAGCTGA
**SmEF1α**	Sm06G02378	SmEF1α-F	ACTGCCCAGGTCATCATCATG
		SmEF1α-R	ACCAGCATCACCGTTCTTCA
**SmTT8**	Sm09G0158	SmTT8-F	GTGATCGTCACACAGAGGCA
		SmTT8-R	CCAACACTACCCTCCACAGT

Supplementary Table 1: Primers for qPCR.
